# Glycerite of Kephaline

**Published:** 1877-06-20

**Authors:** C. G. Polk

**Affiliations:** Philadelphia


### GLYCERITE OF KEPHALINE.

By C. G. POLK, M. D., Philadelphia.

The interest bestowed upon the various preparations of phosphorus in the last few years is important. It is believed that it subserves a high purpose in the animal economy, and exerts a modifying influence upon physiological and pathological conditions in animal organisms, consequently the integrity of organs and functions seem to depend upon an adequate supply and proper formula.

To meet these requisitions, the skill of the chemist has been exhausted, and in vain. The use of the laboratory compounds have been attended with very uncertain advantage.

Andre Sanson says: "The phosphates that are manufactured in the laboratory are not such as should be used, because their form does not allow of their digestion, and assimilation."

Dr. Tillbury Fox, the distinguished writer on skin diseases, says: "There is something essentially special in the organized phosphates, in fact, that have been formed by passing through a living organism, as compared with those artificially prepared. It is not the *amount, but the kind*, which produces the good result."

Dr. Percy, in his essay on phosphorus, for which the prize essay was awarded by the American Medical Association in 1872, says: "That phosphates that enter the animal system, as laboratory compounds, do not perform the same function as the phosphates which enter the system through the natural chemical elaboration of vegetable life."

The above quotations explain why the different preparations of the oxides of phosphorus have been unsatisfactory as remedial agents; they are laboratory compounds, incapable of assuming organismal or nutrient properties. They may prove tonics, but even as tonics they are inferior to iron, quinia and cod liver oil. Phosphorus in the form of the metalloid, taken into the system, is scarcely oxidized at all; only a very minute quantity being transformed into hypophosphorous acid, and if continued for any considerable length of time, Dr. Percy says it induces Bright's Kidney (Nephite Albuminuria). Churchill's Hypophosphites have not sustained his claim as a restorer of phosphorus in an oxidizable form to the system.

Believing that the scientific and rational formula of phosphorus could only be found in a vegetable or an animal organism, I began my experiments in 1857, while a medical student in the University of New York, to form a combination identical with the phosphoids of the brain. This I realized in protagon, an agent now undergoing the ordeal of the medical profession, and which promises to become an extensively used and an esteemed agent.

The subject of this paper differs from protagon in containing only the brain hypophosphites to the exclusion of the phosphites, phosphates and oleo hypophosphrous. I here give its formula:

| | |
|---|---|
| R—Hypophosphite of ammonium | 6 parts. |
| Hypophosphite of calcium | 8 parts. |
| Hypophosphite of sodium | 3 parts. |
| Hypophosphite of magnesium | ½ part. |
| Hypophosphite of manganese | ½ part. |
| Phosphide of nitrogen | 1 part. |
| Tribasic hypophosphorous acid (50 per cent.) | 5 parts. |
| Pure glycerine | 76 parts. |

I pursue, very nearly, Bennett's method in isolating the brain phosphoids. Ten drops of this combination, given three times a day, is a powerful nerve tonic, and an invaluable nerve food. Given with Lœflund's malt extract, I have succeeded far better with my consumptives than I did when I relied mainly on cod liver oil. In fact, my experience has been that, in the earlier stages, this treatment will arrest the disease in a large per cent. of cases, but it will not cure the disease in more than five to eight per cent. of cases after tubercular softening has begun. I believe in the pretubercular stage, it is almost a specific, but it is almost useless to give it in the last stages, with the expectation of immediate restoration to health. It can not restore a ruined lung ; it can not, invariably arrest the ulcerative stages of phthisis; but, yet, my experience justifies me in trusting it with a greater confidence in every stage of phthisis than I can accord to cod liver oil, or any agent in the list of therapeutics.

In practice, nervous diseases furnish a large, interesting, and intricate part of the cases with which the physician has to deal.

Loss of memory, nervous prostration, sexual debility, receive decided benefit from the use of the glycerite of kephaline. If there be a remedy entitled to the name of specific, it belongs to this in the cure of these disorders. My experience has been extensive with it, and the results are generally gratifying. In paresis cerebri, it is the only agent which promises any benefit. It will arrest the progress of locomotor ataxia in a large per cent. of cases; although, it does not restore the nerve structure, which has been already damaged; it usually prevents further extension in that class of cases, which seem to have their origin in sexual excesses.

But, perhaps, of all the diseases, in which I have used the glycerite of kephaline, there is none in which the use is more positive, and unequivocal, than in uterine irritation, evincing itself in the protian forms of hysteria; a class of patients who are usually inveterate and annoying, defying the usual remedies of the materia medica, and breaking our repose in many a weary night. These persons are frequently dyspeptic, and of a low vital standard. They need a powerful nerve tonic, and find it in this agent. I have now, a record of twenty of those cases, in which permanent relief has been secured by the use of this agent.

In infantile diseases, as we would, a driori, have anticipated, glycerite of kephaline is efficacious. Most of the diseases immediately associated with dentition, have their prime factor in a deficiency of the phosphoids, in consequence, the phosphites of magnesium and calcium are not supplied to meet the requirements of the system, in the production of teeth, bones, and in the process of nutrition, irritation is set up in the nervous centres, expending itself upon the brain, lungs, or intestinal canal. By supplying the phosphoid principles required, and not previously present, it relieves dental irritation effectually; for this purpose, I prefer a wine of wheat, made by isolating the phosphoid principles from four pounds of wheat, by heated alcohol, precipitating at a low temperature, dissolving with orthophosphoric acid, and mixing with one pint of port wine. Protagon, or the solution of all the phosphoid principles of brain, in hypophosphorous acid and glycerine, is to be preferred, in infantile cases, to the glycerite of kephaline.

I have here tried to define the therapentical position of this agent without exaggeration, or enthusiasm, and submit these conclusions to the medical profession, conscious that whatever error I have presented will be realized and rejected, and if the agent be as valuable as I think it is, it will eventually receive all the appreciation that is due it.

